# Human Cerebrospinal Fluid Promotes Neuronal Viability and Activity of Hippocampal Neuronal Circuits *In Vitro*

**DOI:** 10.3389/fncel.2016.00054

**Published:** 2016-03-04

**Authors:** Marta Perez-Alcazar, Georgia Culley, Tim Lyckenvik, Kristoffer Mobarrez, Andreas Bjorefeldt, Pontus Wasling, Henrik Seth, Frederik Asztely, Andrea Harrer, Bernhard Iglseder, Ludwig Aigner, Eric Hanse, Sebastian Illes

**Affiliations:** ^1^Institute of Neuroscience and Physiology, The Sahlgrenska Academy, University of GothenburgGothenburg, Sweden; ^2^Department of Neurology, Christian-Doppler-Klinik, Paracelsus Medical UniversitySalzburg, Austria; ^3^Department of Geriatric Medicine, Christian-Doppler-Klinik, Paracelsus Medical UniversitySalzburg, Austria; ^4^Institute of Molecular Regenerative Medicine, Paracelsus Medical UniversitySalzburg, Austria; ^5^Spinal Cord Injury and Tissue Regeneration Center Salzburg (SCI-TReCS), Paracelsus Medical UniversitySalzburg, Austria

**Keywords:** human cerebrospinal fluid, hippocampal neuronal function, hippocampal neuronal survival, organotypic hippocampal slice cultures, hippocampal neuronal cultures, multi-electrode array technology

## Abstract

For decades it has been hypothesized that molecules within the cerebrospinal fluid (CSF) diffuse into the brain parenchyma and influence the function of neurons. However, the functional consequences of CSF on neuronal circuits are largely unexplored and unknown. A major reason for this is the absence of appropriate neuronal *in vitro* model systems, and it is uncertain if neurons cultured in pure CSF survive and preserve electrophysiological functionality *in vitro*. In this article, we present an approach to address how human CSF (hCSF) influences neuronal circuits *in vitro*. We validate our approach by comparing the morphology, viability, and electrophysiological function of single neurons and at the network level in rat organotypic slice and primary neuronal cultures cultivated either in hCSF or in defined standard culture media. Our results demonstrate that rodent hippocampal slices and primary neurons cultured in hCSF maintain neuronal morphology and preserve synaptic transmission. Importantly, we show that hCSF increases neuronal viability and the number of electrophysiologically active neurons in comparison to the culture media. In summary, our data indicate that hCSF represents a physiological environment for neurons *in vitro* and a superior culture condition compared to the defined standard media. Moreover, this experimental approach paves the way to assess the functional consequences of CSF on neuronal circuits as well as suggesting a novel strategy for central nervous system (CNS) disease modeling.

## Introduction

The interstitial fluid (ISF) of the brain parenchyma encompasses all brain cells, and thereby, represents the physiological environment of neurons *in vivo*. The cerebrospinal fluid (CSF), which fills the brain’s ventricles and subarachnoid space, communicates with the ISF via diffusion, since a barrier between the ventricular system and the brain parenchyma is absent in the adult brain (Agnati et al., 1995; Veening and Barendregt, 2010). The concept of volume transmission is based on the distribution of functionally relevant neuroactive substances by the CSF and ISF throughout the central nervous system (CNS; Agnati et al., 1995). A plethora of literature has suggested that CSF-derived molecules induce cellular responses in the brain parenchyma resulting in functional and behavioral consequences (Gato et al., 2005; Bachy et al., 2008; Veening and Barendregt, 2010). How the CSF directly influences neuronal activity and communication in the healthy and diseased brain, however, is far from being understood. So far, our current knowledge is based on electrophysiological characterization of neuronal circuits in acute hippocampal brain slices (Björefeldt et al., 2015), primary brain tissue (Otto et al., 2009) or mouse embryonic stem cell-derived neuronal cultures (Otto et al., 2009) exposed to human CSF (hCSF) for minutes or a few hours. For example, by using multi-electrode array (MEA) recordings in neuronal cultures, we have shown that hCSF has an acute (<15 min) impact on neuronal networks by increasing the activity and the synchronicity (Otto et al., 2009). Recently we confirmed this hCSF-mediated increase in neuronal activity by performing patch-clamp recordings in acute rat hippocampal slices. Moreover, hippocampal CA1 pyramidal neurons in hCSF displayed *in vivo*-like functional properties, which is not seen in routinely used artificial CSF (aCSF). We also provided (first) evidence that these neuromodulatory effects were mediated by G-protein coupled receptors (Björefeldt et al., 2015).

It is, however, unknown how prolonged *in vitro* exposure of hippocampal neurons to CSF influences the viability and function of neurons. Currently used *in vitro* models comprise the cultivation of isolated whole brains or specific brain regions as well as dissociated neuronal cells obtained from rodent brains or human pluripotent stem cells. In order to support survival and functionality of cultured neural cells, different types of cultivation media are used, such as DMEM or Neurobasal/B27-based culture media. Such chemically defined culture media represents, however, an artificial environment for neuronal cells, posing questions about the physiological relevance of the data collected. Moreover, recent works demonstrate that culture media such as Neurobasal or DMEM/F12 suppress neuronal activity (Bardy et al., 2015) and may even be toxic to mature neurons (Hogins et al., 2011; Maggioni et al., 2015). Since the composition of the ISF and CSF within the brain is not completely characterized, it is most likely that all currently used chemically defined media-based approaches are insufficient to create a brain-equivalent physiological environment *in vitro*.

Experimental evaluation of neuronal viability and function after prolonged exposure to CSF is a fundamental prerequisite in order to investigate the influence of CSF on neuronal circuits and for the development of optimized culture conditions for neurons *in vitro*.

In this article, we present an *in vitro* approach, which directly assesses these issues. First, we exposed mature rat hippocampal neuronal cultures to hCSF in order to examine survival and gross morphological alterations in comparison to Neurobasal-A (Nb-A) treated hippocampal neurons. In addition, we applied multielectrode array recordings to characterize the neuronal network properties of hCSF-treated mature hippocampal neurons. In a second set of experiments, we validated the impact of hCSF on neuronal viability and synaptic function in organotypic hippocampal slice cultures from the rat.

## Materials and Methods

### Preparation of Hippocampal Neuronal Cultures on MEAs

Hippocampal neuronal cells were prepared from postnatal (P0–3) Fischer 344 rats (Charles River Deutschland GmbH, Germany) according to published protocols (Brewer, 1997). Neurons were plated at a density of 40,000/well onto poly-D-lysine (1 mg/ml) and laminin (5 μg/ml)-coated (Sigma-Aldrich, both) six-well MEAs, cultured in Nb-A-based media (Nb-A supplemented with B27), Gentamycin (endc. 10 μM) and Glutamine (endc. 200 μM, all Gibco) in a humidified atmosphere (5% CO_2_/95% air) at 37°C. Culture medium was replaced twice a week. Neuronal cultures cultivated for 26–28 days *in vitro* (div) were used for all experiments.

### MEA Measurements and Experimental Design

Baseline activities of hippocampal neuronal cultures were recorded in cultivation media prior to application of either pure hCSF or fresh culture media. According to the number of spike detecting electrodes, the appearance or absence of population bursts (PBs), neuronal cultures were split in two groups. Each well of a six-well MEA contains nine planar electrodes, allowing it to record spontaneous activity of neurons which are in close vicinity (50–100 μm) of the recording electrode. High-active networks are characterized by 90–100% (8–9 out of 9) electrodes detecting spike activity showing partial or synchronous activity. Low-active neuronal networks are characterized by 10–70% (1–6 out of 9) electrodes detecting spike activity showing asynchronous, partial or synchronous activity. To investigate the influence of prolonged hCSF exposure on high- and low-active hippocampal neuronal cultures, cultivation media was removed and substituted with 100 μl pure hCSF/well. The six-well MEAs were then placed back into the incubator. For control experiments 100 μl Nb-A-based media was used. After 24, 48 and 72 h, MEA-recordings were performed to measure the electrophysiological activity of neuronal populations exposed either to hCSF or Nb-A-based media. Each well contained a square grid of nine planar Ti/TiN electrodes (30 μm diameter, 200 μm spacing). Signals from all 54 (6*9) electrodes were simultaneously sampled at 25 kHz, visualized and stored using the standard software MC_Rack provided by Multi Channel Systems. Spike and burst detection was performed offline by custom-built Software (Result, Düsseldorf, Germany). Detailed description of analysis procedures are described elsewhere (Illes et al., 2009, 2014; Hedrich et al., 2014).

### Statistical Analysis of MEA-Data

Data of spikes/2 min, number of PB, PB half width values and number of active electrodes were either analyzed by paired (before and after treatment) or unpaired (different treatments) *t*-test, respectively. Significance level was set at *p* < 0.05. Data are presented as mean ± standard deviation (SD); *n* refers to the number of individual cultures treated either with hCSF or culture media. All statistical data analyses have been performed with GraphPad Prism, version 5.01. Stars indicates significant values (*p < 0.05; **p < 0.01; ***p < 0.001).

### Preparation of Hippocampal Brain Slices

Wistar rats 7–10 days old (P7–10) were used for this study. The animals were housed under a 12 h light/dark cycle with food and water *ad libitum*. All experiments were performed in accordance with the guidelines of the local ethical committee for animal research at the University of Gothenburg and were conducted in accordance with European Union directives on animal rights.

Rats were anesthetized with isofluorane and immediately decapitated. The brain hemispheres were isolated and transferred to ice cold solution (4°C) containing (in mM): 219 Glycerol, 2.5 KCl, 1.2 NaH_2_PO_4_, 1.2 CaCl_2_, 7 MgCl_2_, 25 NaHCO_3_, and 11 Glucose. The solution was bubbled with carbogen gas (95% O_2_ and 5% CO_2_) in order to maintain the pH (7.4). Sagittal slices (300 μm) were cut using a microtome (Microtome HM 650 V, Thermo Fisher Scientific, Loughborough, UK). Slices were maintained in a Hibernate-A media (A12475-01, Gibco, Life Technologies, Sweden) at 4°C until transferred to the membrane. Slices were transferred to a membrane with hCSF and Nb-A-based culture media respectively. Slices were incubated under sterile conditions at 37°C with 5% CO_2_ and 90% humidity. Culture medium was changed at regular time intervals of 2–3 days up to 9 div.

### Organotypic Hippocampal Slice Culture

Organotypic hippocampal slices were prepared in accordance with previously described methods (Stoppini et al., 1991), with slight modifications (Finley et al., 2004; Hogins et al., 2011; Jantzen et al., 2013). A 30 mm diameter, sterile, porous (0.4 μm), transparent and low-protein-binding membrane (Millicell-CM, Millipore catalog # PICM 0RG 50) was used as support for the slices.

Slices were cultured either in hCSF or in a standard medium: Nb-A (10888-022, Gibco, Life Technologies, Sweden), supplemented with B27 and L-glutamine, according to the manufacturer protocol. Antifungal agent, Nystatin, and antibiotics, Penicillin and Streptavidin (P/S), were added to both Nb-A and hCSF. Slices from the same animal were alternately placed on a membrane into wells containing culture media or hCSF.

### hCSF Collection

hCSF samples (age > 60 years, female: 4, male: 4) used for neuronal culture experiments were obtained via lumbar puncture at the Christian-Doppler-Klinik in Salzburg, Austria. Samples came from individuals admitted to the hospital and were collected for diagnostic purposes. hCSF samples were considered normal hCSF when routine laboratory CSF parameters (i.e., basic ion concentration, leukocyte count, number of erythrocytes (if any), total protein, glucose, lactate, CSF cytology and CSF/serum albumin quotient) were in a normal range, and when inflammatory, neurodegenerative, ischemic, CNS neoplastic, or infectious processes were excluded by clinical examinations. The use of hCSF samples for research purposes was approved by the ethics board of the Land Salzburg (415-E/1160/4-2010).

Pooled hCSF (consisting of 5–30 anonymized samples) obtained from patients which had been diagnosed with normal pressure hydrocephalus (NPH) was used for organotypic hippocampal slice cultures. The clinical procedure conformed to the declaration of Helsinki and was performed by means of lumbar puncture by neurologists at the Sahlgrenska University Hospital in Gothenburg, Sweden.

All CSF-samples were handled according to an international accepted consensus guideline (Teunissen et al., 2011). hCSF-samples were routinely centrifuged (2000 g, 10 min) within 2 h after the lumbar puncture to remove cells. Supernatants were aliquoted and stored at −80°C.

### Electrophysiological Recordings from Organotypic Slices

After 9 div, hippocampal slices were placed in a submersion recording chamber and perfused at a constant flow (~2 ml/min) with aCSF containing (in mM): 129 NaCl, 3 KCl, 2 CaCl_2_, 1 MgCl_2_, 26 NaHCO_3_, 1.24 NaH_2_PO_4_ and 10 D-glucose. The aCSF was continuously bubbled with 95% O_2_ and 5% CO_2_ (pH ~7.4) and kept at room temperature (20–22°C). Whole-cell patch-clamp recordings were performed for at least 5 min in CA1 pyramidal cells, which were visually identified using infrared differential interference contrast video microscopy mounted on a Nikon Y-QT (Japan) microscope. The intracellular solution contained (in mM): 95 Cs-methanesulfonate, 2 NaCl, 10 HEPES, 5 QX-314, 4 Mg-ATP, 0.4 GTP, 15 phosphocreatine and 20 BAPTA (pH ~7.3, 280–305 mOsm/kg). Patch pipette resistance ranged from 3–5.5 MΩ.

Miniature excitatory postsynaptic currents (mEPSCs) were recorded at −70 mV in the presence of 0.5 μm tetrodotoxin (TTX) and 0.1 μm picrotoxin (PTX), using an EPC-9 amplifier (HEKA Elektronik, Lambrecht, Germany). mEPSCs were sampled at 10 kHz, filtered at 2.9 kHz, and the first 100 mEPSCs were analyzed blinded using the Software Mini-Analysis (version 5.1.4; Synaptosoft, Decatur, GA, USA).

### Histological Evaluation: Nissl Staining of Organotypic Hippocampal Slice Cultures

Cresyl violet staining was used for histological evaluation of organotypic slices after 9 div. Slices attached to a small rectangle of the supporting membrane were cut from the whole membrane insert, fixed with 4% paraformaldehyde at 4°C (24 h) and washed three times in phosphate-buffered solution (PBS) for 5 min. Slices were mounted on Superfrost Ultra Plus slides (Menzel-Gläser, Germany) and dried overnight. Slices were hydrated using decreasing concentrations of ethanol (90, 70, 50%, 1 min in each concentration), rinsed twice with water for 2 min and stained with 1% Cresyl violet for 5 min at 60°C. Slices then were dehydrated using increasing concentrations of ethanol (50, 70, 90%, 1 min in each concentration) and xylene and mounted with DPX mounting media (Sigma-Aldrich, Germany).

### Morphological Analysis of Organotypic Hippocampal Slices

Morphological analysis was carried out using an Axio Observer microscope (Zeiss, Germany) objective NA 0.3 and camera NA 0.5. Area measurements of the organotypic hippocampal slices stained with Cresyl violet were performed using ImageJ Software (NIH) from images taken under 10× magnification. The width of the stratum pyramidale was measured as the average width over 500 μm from organotypic hippocampal slice cultures in which it was possible to clearly define the cell body layer. The analysis was performed blinded with regard to the experimental group.

### Viability Analysis of Organotypic Hippocampal Slices

To measure the viability of the cultured hippocampal slices, 80 μl of CellEvent Caspase-3/7 Green ReadyProbes (Life Technologies Europe BV, Stockholm, Sweden) was added to 500 μl of culture media (Nb-A or hCSF) and the preparations were incubated for 30 min at 37°C (95% O_2_, 5% CO_2_). Slices were then fixed by incubation in HistoFix (Histolab Products AB, Göteborg, Sweden) for 1 h. Preparations were mounted on glass slides using 40–60 μl ProLong^®^ Gold Antifade supplemented with DAPI nuclear stain (Life Technologies Europe BV, Stockholm, Sweden). Slides were stored at 4°C. An Olympus DP72 microscope equipped with an Olympus U-RFL-T mercury lamp was used for digital micro-imaging; the digital image analysis was captured with Olympus CellSens Dimension. Final analysis was performed in ImageJ (NIH, Bethesda, MD, USA). For every preparation three independent areas in the CA1 were selected and the intensity measured in gray scale. Intensities were averaged and normalized to the DAPI intensity to control for any difference in exposure. The CellEvent Caspase-3/7 Green ReadyProbes was evaluated prior to the experiments by comparing untreated controls to hippocampal slices exposed to 150 μM NMDA for 1 h.

### Statistical Analysis of Organotypic Hippocampal Slice Culture Experiments

Statistical significance for immunohistochemical analysis was assessed using Mann-Whitney test. Significance level was set at *p* < 0.05. All data are presented as mean ± SD. All the analysis was performed using GraphPad Prism (version 5.02).

## Results

### Human Cerebrospinal Fluid Enhances Neuronal Function in Hippocampal Neuronal Cultures Within 72 h

**Figure 1:**
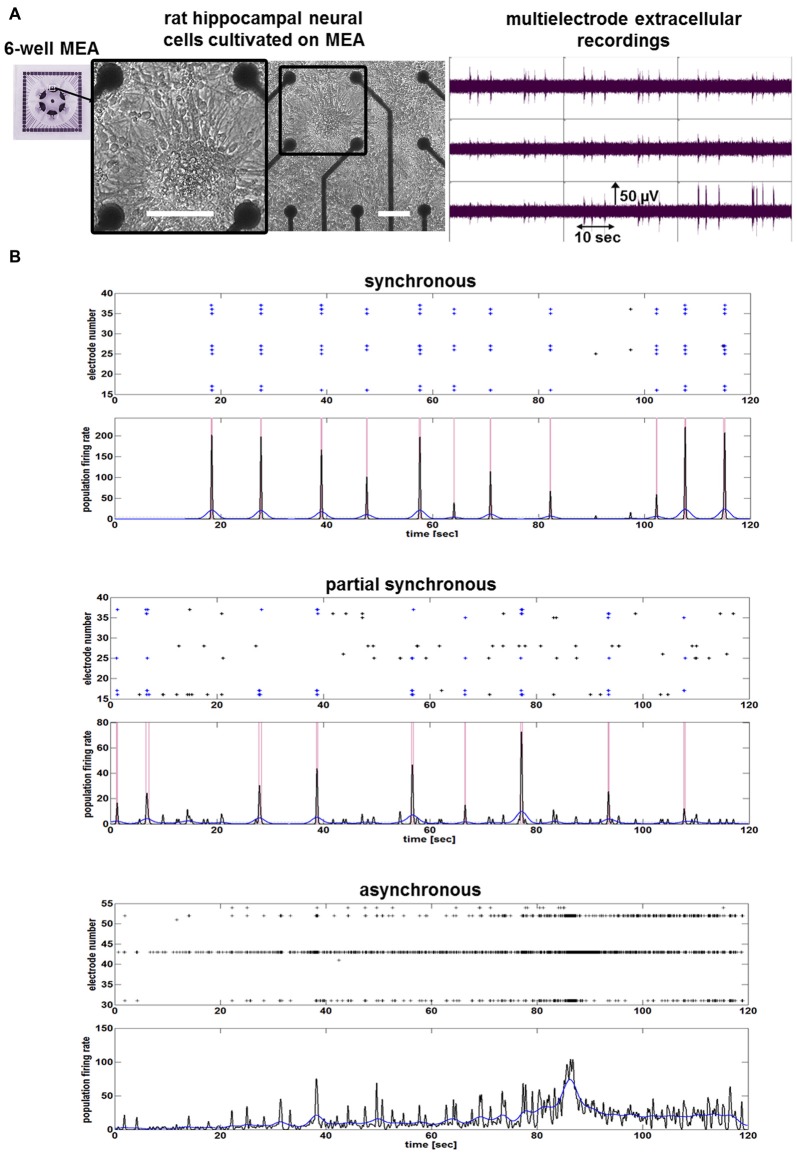
**Neuronal network activity of mature rat hippocampal neuronal cultures. (A)** Images show morphology and neuronal network activity of rat hippocampal neuronal cells cultivated on a nine-electrode array (28 div) of a six-well multi-electrode array (MEA). White bars represent scale bars (100 μm). Image with black frame shows detailed morphology of neuronal cells cultivated on MEA. **(B)** Examples of spike raster plots and population firing ratesillustrate the activity of one synchronous, one partial synchronous and one asynchronous rat hippocampal neuronal network.

To assess the chronic influence of hCSF on neuronal function of hippocampal neurons *in* *vitro*, we characterized their baseline electrical activity. Cultures consisted of spontaneously active (as determined by electrophysiology) mature neurons (Figure 1). MEA recordings show that mature hippocampal neuronal networks are either asynchronous (absence of population bursting), partially synchronous or synchronous active (Figure 1). After the baseline neuronal activity in the cultivation media was determined, we replaced the media with pure hCSF samples. Afterwards, neuronal cultures were placed back into the incubator and neuronal network activities were measured 24, 48 and 72 h after application of hCSF.

**Figure 2:**
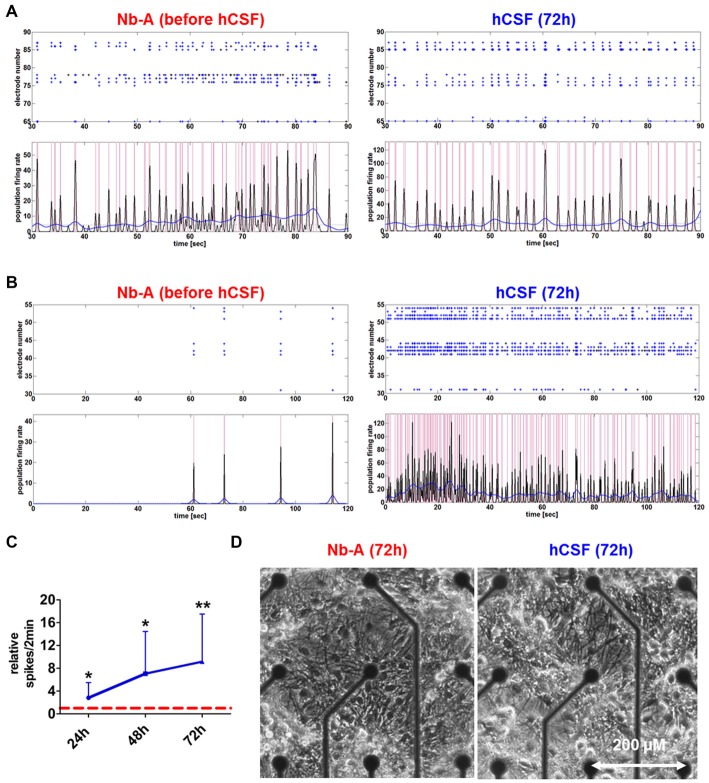
**Network activities and morphology of hippocampal neurons after prolonged exposure to human CSF (hCSF). (A)** Examples of spike raster plotsand population firing ratesillustrate the activity of high-active mature hippocampal neurons (4 weeks cultured in Neurobasal-A (Nb-A) medium) before and after (72 h) exposure to hCSF. **(B)** Examples of spike raster plotsand population firing ratesillustrate the activity of low-active mature hippocampal neurons (4 weeks cultured in Nb-A medium) before and after (72 h) exposure to hCSF. **(C)** Diagram illustrates the relative change of spiking activity after the application of hCSF to high- and low-active hippocampal neuronal cultures (blue line) in relation to baseline activity recorded in cultivation media (red dotted line) (mean ± SD, 6 hCSF-samples used in 12 neuronal cultures obtained from three independent rat preparations). **(D)** Morphology of mature rat hippocampal neuronal cells (28 div) 3 days after the application of either fresh Nb-A-based media or hCSF. *p < 0.05; **p < 0.01.

Morphological examination did not reveal obvious differences between hippocampal neuronal cultures exposed to hCSF and fresh culture media for 3 days (Figure 2D). hCSF-treated hippocampal neuronal networks (*n* = 12, 8 hCSF obtained from different individuals were used and each sample were applied to 1–2 neuronal networks) showed a gradual increase in spiking activity in comparison to baseline activity (Figure 2). More specifically, hCSF increased spiking activity up to 2.8 ± 1.8-fold (*p* = 0.03, mean ± SD) after 24 h, 7.0 ± 6.0-fold (*p* = 0.01, mean ± SD) after 48 h and 9.1 ± 8.1-fold (*p* = 0.006, mean ± SD) after 72 h of hCSF incubation (Figure 2C). Morphological examination did not reveal obvious differences between hippocampal neuronal cultures exposed to hCSF and fresh culture media for 3 days each (Figure 2D). Since the degree of spontaneous electrical activity of the networks varied between neuronal populations, leading to substantial variation of neuronal parameters, we sub-classified hippocampal networks in high- and low-activity categories. Sub-classification was performed according to the mean spiking activity, the number of spike detecting electrodes and the appearance or absence of PBs. High-active networks were characterized by a mean spiking activity >150 spikes/2 min, with 90–100% (8–9 out of 9) of electrodes detecting spikes and showing partial or synchronous activity (Figure 2A). Low-active neuronal networks were characterized by <150 spikes/2 min, with 10–70% (1–6 out of 9) of electrodes registering spikes and showing asynchronous, partial or synchronous activity (Figure 2B). Neuronal cultures showing less than 20 spikes/2 min were excluded.

**Figure 3:**
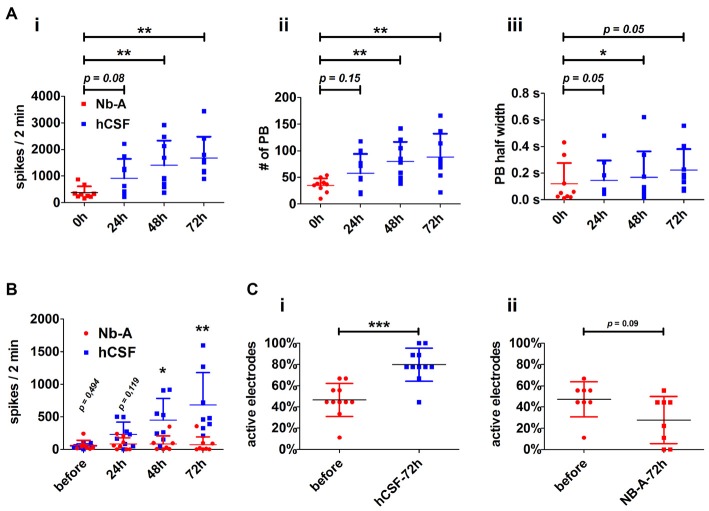
**Neuronal paramters describing the functional impact of hCSF on hippocampal neuronal network activity. (A)** Diagrams illustrate the course of neuronal network activity after the application of hCSF to high-active hippocampal neurons indicated by the mean number of spikes **(i)**, number of population bursts (PBs) **(ii)** and PB half width (PB half width) **(iii)**. Each dot represents the impact of one individual CSF sample on individual hippocampal neurons (mean ± SD, 5 hCSF-samples tested in six individual neuronal cultures obtained from three independent rat preparations). **(B)** Diagrams illustrate the mean number of spikes of low-active hippocampal neurons treated either with hCSF (blue) or fresh Nb-A-based media (red) for 3 days. Each dot represents the impact of one individual hCSF-sample on individual hippocampal neurons cultivated in one well of a six-well MEA (mean ± SD, 4 hCSF-samples tested in eight individual neuronal cultures obtained from two independent rat preparations). **(C)** Diagrams illustrate the percentage of activity detecting electrodes in hippocampal neuronal networks before and 72 h after treatment with hCSF (blue) or fresh Nb-A-based media (red) (mean ± SD, 6 hCSF-samples tested in 11 individual neuronal cultures obtained from three independent rat preparations). *p < 0.05; **p < 0.01; ***p < 0.001.

Both high- and low-active hippocampal neuronal cultures showed a gradual increase in network activity within 72 h as indicated by increased spiking activity (high-active neuronal cultures: media 396.5 ± 245 spikes/2 min vs. hCSF 1592 ± 801.9 spikes/2 min; *p* = 0.0016, low-active neuronal cultures: media 50 ± 36 spikes/2 min vs. hCSF 682 ± 493 spikes/2 min; *p* = 0.007, *t*-test), population bursting (PB; high-active neuronal cultures: media 36 ± 13 PB vs. hCSF 97 ± 39 PB; *p* = 0.0059, both *t*-test) and PB half width (high-active neuronal cultures: media 0.080 ± 0.109 s vs. hCSF 0.181 ± 0.105 s; *p* = 0.05, *t*-test; Figure 3). In addition, low-active hippocampal neuronal cultures cultivated for 3 days in hCSF converted into high-active ones (media 50 ± 36 spikes/2 min vs. hCSF 682.4 ± 493.5 spikes/2 min, *p* = 0.004, *t*-test) in which nearly all electrodes detected spiking activity (media 48 ± 17% out of nine electrodes vs. hCSF 84 ± 11% out of nine electrodes, p < 0.001, *t*-test; Figure 3Ci). In contrast, this increase in spiking activity and number of active electrodes was absent in hippocampal neuronal cultures exposed to fresh culture media (media 77.5 ± 83 spikes/2 min, 47 ± 16% out of nine electrodes vs. fresh media 66.2 ± 35 spikes/2 min, 27 ± 22% out of nine electrodes, *p* = 0.932, *t*-test) (Figure 3Cii).

### Human Cerebrospinal Fluid Enhances Neuronal Viability and Preserves Synaptic Transmission in Hippocampal Organotypic Slice Cultures

**Figure 4:**
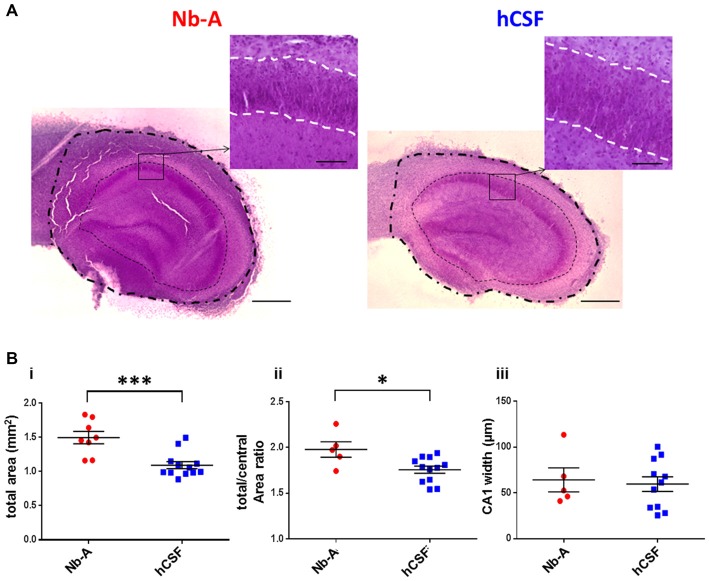
**Gross morphology of slice cultures in Neurobasal-A-based medium and in hCSF. (A)** Representative images of Nissl stained organotypic hippocampal slices incubated 9 div with Nb-A (left image) and hCSF (right image). The *outer* dashed/dotted line delineates the border for the total area measurement, and the *inner* dotted line delineates the border for the central area measurement. Small inserts at higher magnification show the pyramidal cell layer in the CA1 region (str. pyramidale) and which is delineated with a white dashed line. Scale bars 500 and 100 μm, respectively. **(B)** Diagrams showing values of the total hippocampal area in mm^2^ **(i)**, total/central area **(ii)**, and the width of CA1 pyramidal cell body layer (stratum pyramidale) **(iii)** for organotypic hippocampal slices cultured in Nb-A (red, *N* = 8) and cultured in hCSF (blue, *N* = 13). *p < 0.05; ***p < 0.001.

To evaluate the feasibility of culturing organotypic hippocampal slices in hCSF we compared organotypic hippocampal slices cultured for 9 days in hCSF with those cultured for the same time in culture media. We first compared the gross morphology of the organotypic hippocampal slices using Nissl staining (Figure 4A). Hippocampal morphology was well preserved in organotypic slices cultured under both conditions. We noted, however, that organotypic hippocampal slices cultured in culture media appeared to be larger than those cultured in hCSF. In particular, peripheral parts outside the cell body layers were expanded in organotypic hippocampal slices cultured in Nb-A. To examine this more closely we quantified three different areas of the organotypic hippocampal slices; the total area, the central area and the area of the CA1 pyramidal cell layer, as schematically illustrated in Figure 1A. The total area was indeed larger in the Nb-A treated group by 36.9% (Nb-A; 1.50 ± 0.25 mm^2^, *N* = 8. hCSF; 1.09 ± 0.18 mm^2^, *N* = 12. *p* = 0.0005, Mann-Whitney U test) in organotypic hippocampal slices cultured in Nb-A compared to slices cultured in hCSF (Figure 4Bi). The increase in the area was not evenly distributed in the organotypic hippocampal slices and mainly involved expansion of the peripheral parts. Thus, when the total area was normalized to the central area (restricted by and including the cell body layers) the slices cultured in Nb-A were still significantly larger by 12.9% (Figure 4Bii, total/central area ratio, Nb-A; 1.98 ± 0.19, *N* = 5. hCSF; 1.76 ± 0.14, *N* = 12. *p* = 0.026, Mann-Whitney U test). In contrast to the larger peripheral area of the organotypic hippocampal slices cultured in Nb-A, there was no significant difference in the width of the CA1 pyramidal cell body layer (str. pyramidale, Figure 4Biii, Nb-A; 64.3 ± 29.3 μm, *N* = 5. hCSF; 59.7 ± 26.7 μm, *N* = 11. *p* = 0.82, Mann-Whitney U test).

**Figure 5:**
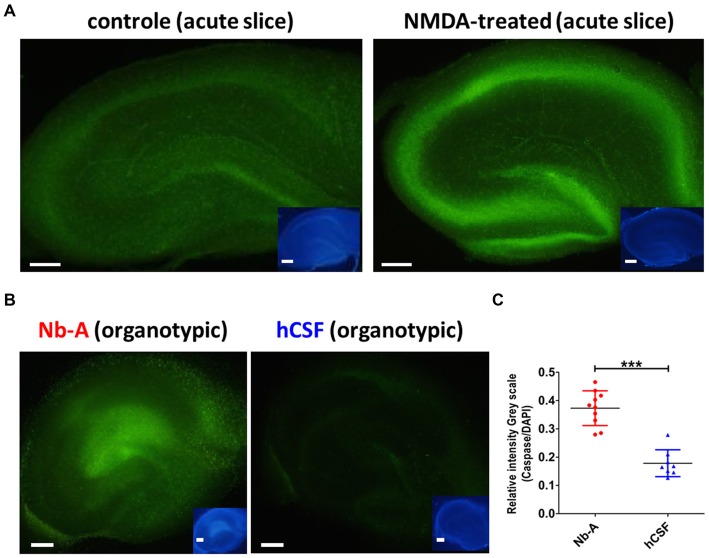
**Expression of the cell death marker caspase-3/7 in organotypic hippocampal slices cultured in Nb-A and in hCSF. (A)** Validation of caspase-3/7 staining as reporter of NMDA-induced cell death in acute hippocampal slices. Examples showing caspase-3/7 staining of an acute hippocampal slice exposed to 150 μM of NMDA for 1 h (left), and a control slice left untreated for 1 h (right). Inserts show the same slices stained with DAPI. **(B)** Examples showing caspase-3/7 staining of an organotypic hippocampal slice cultured in Nb-A for 9 days (left), and in hCSF for 9 days (right). Insets show the same slices stained with DAPI. **(C)** Diagram showing average intensity of caspase-3/7 staining (the ratio of Caspase-3/7 intensity to DAPI intensity, in gray scale) in organotypic hippocampal slices cultured in Nb-A (red, *N* = 10, 0.37 ± 0.06) and in hCSF (blue, *N* = 8, 0.17 ± 0.04). Intensities were measured in the CA1 area at three independent locations per preparation and averaged (mean ± SD). ***p < 0.001.

To compare the viability of organotypic hippocampal slices cultured in hCSF and Nb-A, we examined the expression of caspase-3/7, which is upregulated in association with neuronal cell death. We first tested the validity of the caspase-3/7 method by exposing acute hippocampal slices to 150 μM NMDA for 1 h, a treatment known to induce excitotoxic cell death (Burguillos et al., 2011). As shown in Figures 5A,C, NMDA treatment induced a clear increase in caspase-3/7 expression as compared with control slices left untreated for 1 h. The small insets showed corresponding nuclear staining from the same slices using DAPI. As exemplified in Figure 5B, and quantified in Figure 5C, organotypic hippocampal slices cultured in hCSF exhibited significantly less expression of caspase-3/7 (normalized to the expression of DAPI in each slice) than organotypic hippocampal slices cultured in Nb-A (0.37 ± 0.06, *N* = 10; hCSF: 0.17 ± 0.04, *N* = 8, *p* < 0.0001, mean ± SD), indicating less apoptotic cell death.

**Figure 6:**
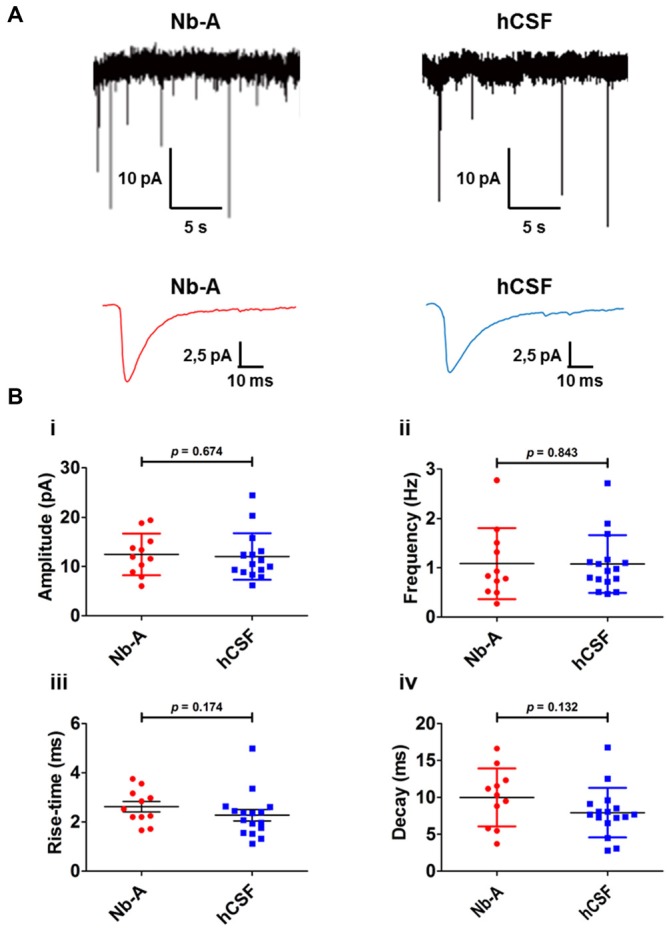
**Miniature AMPA receptor-mediated transmission (AMPA mEPSCs) in organotypic hippocampal slices cultured in Nb-A and in hCSF. (A)** Example recordings of miniature AMPA receptor-mediated EPSCs in CA1 pyramidal cells from organotypic hippocampal slices cultured in Nb-A (left) or in hCSF (right). Corresponding average AMPA mEPSCs are shown below. **(B)** Diagrams showing amplitude **(i)**, frequency **(ii)**, rise-time **(iii)** and decay **(iv)** of AMPA mEPSCs recorded in organotypic hippocampal slices cultured in Nb-A (*N* = 11) or in hCSF (*N* = 16; mean ± SD).

As a functional comparison between organotypic hippocampal slices cultured in hCSF or Nb-A, we used whole-cell patch-clamp recordings of miniature AMPA receptor mediated EPSCs (mEPSCs) from CA1 pyramidal cells (Figures 6A,B). We found that glutamatergic synaptic transmission was preserved under both culture conditions, and no significant differences were observed in amplitude (Figure 6Bi, Nb-A; 12.5 ± 4 pA, *N* = 11. hCSF; 12.1 ± 5 pA, *N* = 16, *p* = 0.67), frequency (Figure 6Bii, Nb-A; 1.09 ± 0.7 Hz, *N* = 11. hCSF; 1.08 ± 0.6 Hz, *N* = 16, *p* = 0.84), rise-time (Figure 6Biii, Nb-A; 2.62 ± 0.7 ms, *N* = 11. hCSF; 2.28 ± 0.9 ms, *N* = 16, *p* = 0.18), or decay-time (Figure 6Biv, Nb-A; 9.99 ± 3.9 ms, *N* = 11. hCSF; 7.93 ± 3.4 ms, *N* = 16, *p* = 0.13) of mEPSCs.

## Discussion

We present a novel experimental approach to assess the influence of hCSF on neuronal circuits. This approach was validated by prolonged exposure of organotypic hippocampal slices and primary neuronal cultures to hCSF in combination with electrophysiology and analysis of cell survival. Our results indicate that hCSF is well tolerated by rodent neurons, providing new possibilities to examine the effects of healthy and pathological hCSF on neuronal function.

We established the six-well MEA as a convenient tool to analyze the functional influence of CSF on neuronal circuits, requiring only small volumes of hCSF (100–200 μl) as well as allowing analysis of several different hCSF samples simultaneously. Our results reveal that hCSF treatment resulted in a robust increase in spontaneous spiking activity and an increased number of electrodes detecting neuronal activity in hippocampal neuronal cultures in comparison to Nb-A media cultivated cultures. This is in line with a recent study demonstrating that Neurobasal media suppresses neuronal activity in cultivated rodent and human neurons (Bardy et al., 2015). Moreover, organotypic hippocampal slice cultures showed a decreased number of apoptotic Caspase-3/7 positive cells when cultured in hCSF as compared to Nb-A. Therefore, hCSF could represent a more physiological environment for *in vitro* cultivated neurons than currently used culture media. The development of chemically defined culture media that increase survival and preserve physiological properties of neurons is an important goal in neuroscience and stem cell research (Livesey, 2015). Therefore, hCSF could serve as a physiological “control medium” when evaluating new artificial cultivation media *in vitro*.

The use of CSF from human individuals in rodent-based *in vitro* model systems was motivated for several reasons. (i) The cultivation of organotypic slices required several milliliters of CSF whereas the CSF volume in a rodent is in the microliter range. Thus, we used pooled hCSF for the cultivation of organotypic slices and individual hCSF samples for hippocampal neurons cultured in six-well MEAs. (ii) Since pathological CSF obtained from patients suffering different types of CNS disease could be used when creating relevant disease models, we were aiming to obtain proof-of-concept evidence that both rodent-based *in vitro* neuronal cultures and organotypic slices tolerate hCSF. We believe that the use of hCSF will be more relevant to predict human brain pathophysiological processes than the use of rodent CSF. However, the volume of CSF routinely collected during the diagnosing of neurodegenerative diseases, such as Alzheimer’s disease, is in a milliliter range. We illustrate that neuronal cells cultured on six-well MEAs can be used to analyze the impact of a limited volume of hCSF. (iii) Human pluripotent stem cell-derived neurons might represent a more appropriate pre-clinical *in vitro* model system to reveal the functional influence of hCSF. On the other hand, the rodent hippocampal neuronal circuit is well characterized and hippocampal slice cultures more reliably represent the *in vivo* hippocampal neuronal circuit than PSC-derived neuronal cultures.

Acute application (15 min to 1 h) of hCSF increases neuronal activity both at a single cell and network level (Otto et al., 2009; Görtz et al., 2013; Jantzen et al., 2013; Björefeldt et al., 2015). In these previous studies, the effect of hCSF on primary cortical and mESC-derived neuronal cultures (Otto et al., 2009) and in acute hippocampal brain slices (Björefeldt et al., 2015) was studied with respect to an aCSF. Interestingly, hippocampal CA1 pyramidal neurons in hCSF displayed lowered firing thresholds, depolarized resting membrane potentials and reduced input resistance, thus mimicking properties of pyramidal neurons recorded *in vivo* (Björefeldt et al., 2015). Physiological electrolyte concentrations, together with yet unidentified supportive factors, are likely to promote the robust neuronal viability and increased neuronal activity observed during long-term cultivation in hCSF. In the future, hCSF-based *in vitro* approaches and CSF-biomarker screening (Zetterberg et al., 2014; Hölttä et al., 2015) could be an efficient combination to identify factors in the CSF with either supportive or deteriorative influence on neuronal function. Insights from such studies could be used to develop more physiological culture media, and thus facilitate the development of more physiologically relevant brain disease models.

## Conclusion

We demonstrate that the cultivation of hippocampal neuronal cultures and organotypic slices in hCSF constitutes a feasible and advantageous approach to studying neuronal activity *in vitro*. hCSF-based *in vitro* model systems may turn out to be the golden standard for evaluating artificial cultivation media. Moreover, our work may pave the way the chronic influence of normal and pathological hCSF on neuronal circuits.

## Author Contributions

MP-A, GC and FA performed the histological evaluations of organotypic slices. GC and AB performed the electrophysiological analysis of organotypic slices. TL and PW prepared organotypic slices cultures. KM and HS performed the capase-3/7 evaluations of organotypic slices. PW, AH and BI collected human CSF, clinical evaluation and CSF analysis for the identification of normal CSF samples. SI performed the MEA-recordings and analysis of hippocampal neuronal networks. LA did critical revision of the manuscript and interpretation of the data. EH and SI conceived the study and wrote the manuscript.

## Conflict of Interest Statement

The authors declare that the research was conducted in the absence of any commercial or financial relationships that could be construed as a potential conflict of interest. SI received financial support by EISAI to conduct research. However the study design, data collection, data interpretation and data presentation were not influenced by EISAI.

## Abbreviations

aCS: artificial cerebrospinal fluid
CSF: cerebrospinal fluid
div: days *in vitro*
hCSF: human cerebrospinal fluid
Nb-A: Neurobasal-A
PB: population burst
mEPSC: miniature excitatory postsynaptic current.
