# Corrigendum: Human Cerebrospinal Fluid Promotes Neuronal Viability and Activity of Hippocampal Neuronal Circuits *In Vitro*

**DOI:** 10.3389/fncel.2016.00227

**Published:** 2016-10-10

**Authors:** Marta Perez-Alcazar, Georgia Culley, Tim Lyckenvik, Kristoffer Mobarrez, Andreas Björefeldt, Pontus Wasling, Henrik Seth, Frederik Asztely, Andrea Harrer, Bernhard Iglseder, Ludwig Aigner, Eric Hanse, Sebastian Illes

**Affiliations:** ^1^Institute of Neuroscience and Physiology, The Sahlgrenska Academy, University of GothenburgGothenburg, Sweden; ^2^Department of Neurology, Christian-Doppler-Klinik, Paracelsus Medical UniversitySalzburg, Austria; ^3^Department of Geriatric Medicine, Christian-Doppler-Klinik, Paracelsus Medical UniversitySalzburg, Austria; ^4^Institute of Molecular Regenerative Medicine, Paracelsus Medical UniversitySalzburg, Austria; ^5^Spinal Cord Injury and Tissue Regeneration Center Salzburg, Paracelsus Medical UniversitySalzburg, Austria

**Keywords:** human cerebrospinal fluid, hippocampal neuronal function, hippocampal neuronal survival, organotypic hippocampal slice cultures, hippocampal neuronal cultures, multi-electrode array technology

Reason for Corrigendum:

In the original article, we have neglected to include Stephan Theiss (Institute of Clinical Neuroscience and Medical Psychology, Medical Faculty, Heinrich Heine University, Düsseldorf, Germany, and Result Medical GmbH, Düsseldorf, Germany). ST developed analyses programs to analyze hippocampal neuronal network activity and interpreted the data. His contribution to this work was supported by the German Ministry of Education and Research (BMBF: FKZ 031B0010B) and the European Union (EURO-TRANS-BIO project In-HEALTH). The authors apologize for this oversight. This error does not change the scientific conclusions of the article in any way.

## Author contributions

MP, GC, and FA performed the histological evaluations of organotypic slices. GC and AB performed the electrophysiological analysis of organotypic slices. TL and PW prepared organotypic slices cultures. KM and HS performed the capase-3/7 evaluations of organotypic slices. PW, AH, and BI collected human CSF, clinical evaluation and CSF analysis for the identification of normal CSF samples. SI performed the MEA-recordings and analysis of hippocampal neuronal networks. LA did critical revision of the manuscript and interpretation of the data. EH and SI conceived the study and wrote the manuscript.

### Conflict of interest statement

The authors declare that the research was conducted in the absence of any commercial or financial relationships that could be construed as a potential conflict of interest. SI received financial support by EISAI to conduct research. However, the study design, data collection, data interpretation and data presentation were not influenced by EISAI.

